# Surveillance of communicable diseases using social media: A systematic review

**DOI:** 10.1371/journal.pone.0282101

**Published:** 2023-02-24

**Authors:** Patrick Pilipiec, Isak Samsten, András Bota

**Affiliations:** 1 Department of Computer and Systems Sciences, Stockholm University, Kista, Sweden; 2 School of Business and Economics, Maastricht University, Maastricht, The Netherlands; 3 Embedded Intelligent Systems Lab, Department of Computer Science Electrical and Space Engineering, Luleå University of Technology, Luleå, Sweden; Binghamton University Thomas J Watson School of Engineering and Applied Science, UNITED STATES

## Abstract

**Background:**

Communicable diseases pose a severe threat to public health and economic growth. The traditional methods that are used for public health surveillance, however, involve many drawbacks, such as being labor intensive to operate and resulting in a lag between data collection and reporting. To effectively address the limitations of these traditional methods and to mitigate the adverse effects of these diseases, a proactive and real-time public health surveillance system is needed. Previous studies have indicated the usefulness of performing text mining on social media.

**Objective:**

To conduct a systematic review of the literature that used textual content published to social media for the purpose of the surveillance and prediction of communicable diseases.

**Methodology:**

Broad search queries were formulated and performed in four databases. Both journal articles and conference materials were included. The quality of the studies, operationalized as reliability and validity, was assessed. This qualitative systematic review was guided by the Preferred Reporting Items for Systematic Reviews and Meta-Analyses (PRISMA) guidelines.

**Results:**

Twenty-three publications were included in this systematic review. All studies reported positive results for using textual social media content to surveille communicable diseases. Most studies used Twitter as a source for these data. Influenza was studied most frequently, while other communicable diseases received far less attention. Journal articles had a higher quality (reliability and validity) than conference papers. However, studies often failed to provide important information about procedures and implementation.

**Conclusion:**

Text mining of health-related content published on social media can serve as a novel and powerful tool for the automated, real-time, and remote monitoring of public health and for the surveillance and prediction of communicable diseases in particular. This tool can address limitations related to traditional surveillance methods, and it has the potential to supplement traditional methods for public health surveillance.

## 1 Introduction

Communicable diseases are a severe threat to public health [1]. These infectious diseases include, among others, dengue, Ebola, malaria, measles, different strains of influenza, and Zika virus. In particular, influenza can result in respiratory symptoms of varying severity, and it can cause high mortality among the vulnerable population of older adults who also have a chronic condition related to the respiratory or immune system [2, 3].

Every year, seasonal influenza causes approximately half a million deaths globally [4, 5]. The 1918 Spanish flu pandemic was estimated to result in the mortality of 40 million people [6]. In addition, communicable diseases can also have catastrophic effects on the economy and society [7]. For example, seasonal influenza was estimated to have a financial burden of $83.3 billion annually in the United States alone [3].

Recently, the large disrupting effect of communicable diseases has again been observed with the outbreak of severe acute respiratory syndrome coronavirus 2 (hereafter SARS-CoV-2), which is assumed to have emerged in the city of Wuhan in China, after which it quickly spread around the globe [8]. Many governments chose a lockdown of society during the outbreak to increase control over this virus, to protect the healthcare system from overload, to mitigate the potential spread and to limit the number of casualties [9]. The International Monetary Fund (IMF) has indicated that this lockdown and the effects of SARS-CoV-2 resulted in a contraction of European economies, by on average, seven percent in 2020 [10].

This illustrates that the context of our society should also be considered. The ongoing fast-paced mobility of people requires a global system for the surveillance of communicable diseases, since the public health in one country can easily and rapidly be impacted that of by another country located on the other side of the planet.

Therefore, there is a need for public health authorities to detect outbreaks of communicable diseases as early as possible, to monitor these diseases and to initiate preventive measures immediately [11–13]. In addition, there exists an ongoing urgency to develop new technologies to forecast communicable disease outbreaks [14–17].

Early detection of communicable diseases is crucial to organize and allocate the required health resources, to control the spread of the disease and to avoid or mitigate further contamination [18]. This urgency is even more significant in the case of epidemic outbreaks, such as the novel SARS-CoV-2 [8], which demand real-time monitoring and rapid initiation of appropriate interventions [19].

However, the traditional methods for public health surveillance have many shortcomings, such as a lag between data collection and reporting [20–22]. To address these drawbacks, a proactive method is needed to automatically detect and monitor disease outbreaks worldwide in real time and to minimize any delays in this process [23, 24].

The emergence and widespread adoption of social media platforms has received a great amount of attention in the literature [25]. People share significant information on health-related experiences on social media [26, 27]. Various studies have indicated that the analysis of health-related content published to social media has the potential to significantly improve the public health surveillance system [28–30]. In addition, in the preceding years, various studies have been published that utilized health-related textual content from social media for the purpose of public health surveillance of communicable diseases. Furthermore, three reviews [31–33] have been performed on the topic of internet-based public health surveillance, and while these reviews provide new insights about the vast opportunities of using social media content for public health surveillance, these reviews are not systematic reviews.

We acknowledge that only five systematic reviews have been conducted thus far that are somewhat related to this topic. First, Velasco et al. [34] found that although incorporating digital content as a source for public health surveillance has great potential, there is a reluctance among public health authorities to include this content in the systems for public health surveillance. Second, Charles-Smith et al. [35] found that analyzing content published to social media has the potential to increase public health, but they based their findings on only 10 publications. Third, Fung et al. [36] performed a systematic review of 12 studies that utilized social media content published during the 2014–2015 Ebola epidemic in West Africa, and they reported that no study evaluated their utility for any public health organization. The aforementioned systematic literature reviews were, among others, not tailored to communicable diseases [34, 35] and emphasized only one regional epidemic [36]. Fourth, Abad et al. [37] conducted a scoping review to summarize the literature on applications of natural language processing for digital public health surveillance, and they emphasized databases in the field of medicine and public health in their literature search strategy. Not including relevant databases in the field of computer science, data science, and information science is a limitation of their study. Fifth, Gupta and Katarya [38] performed a systematic review on the utilization of social media data in real-time public health surveillance systems, and they concluded that, compared to traditional methods, the analysis of social media data has increased the ability of these systems to predict diseases. However, two differences of their systematic review are that the literature search involved all types of artificial intelligence instead of focusing on the branch of natural language processing. As a consequence, they had a limited emphasis on the technical aspects of natural language processing in detail, such as explaining how preprocessing of natural language was performed and which methods and tools were used. We believe that a new systematic review is required that emphasizes the technical aspects of these applications of natural language processing for the surveillance of communicable diseases. In addition, considering the changes in social media use and advances in the respective fields of science in the foregoing half decade since the reviews above were published, some of these reviews may already be outdated.

Therefore, in this paper, we perform a new and thorough systematic literature review that investigates how textual content published to social media can be used for the purpose of the surveillance and prediction of communicable diseases. A systematic review of the evidence on this topic can greatly benefit public health authorities. In addition to evidence about the effectiveness of specific methods, this systematic review also provides a synthesis of the communicable diseases that were studied, social media platforms that were used, and which software and algorithms were utilized in these studies. If textual content from social media can indeed be used to surveille and predict outbreaks of communicable diseases, then such systems may become a powerful tool and asset for public health authorities and have the potential to address most of the limitations of the methods that are commonly used in traditional public health surveillance systems [28–30].

There is an opportunity to develop a proactive global public health surveillance system [23]. This tool should enable the automated and real-time monitoring of diseases worldwide by including information from various novel sources containing contextual information about social media users while minimizing the overall processing time from data collection to the reporting of identified findings [24]. This tool could significantly benefit rapid and evidence-based decision-making regarding infectious disease outbreaks [24]. A systematic review could provide more insight into this opportunity.

## 2 Background

Public health surveillance, also called epidemiologic surveillance, involves the ongoing and systematic collection, management, and monitoring of data about diseases, with the purpose of identifying trends, e.g., [39–41]. The overall objective of public health surveillance is to detect outbreaks of diseases at the earliest possible time so that the required preparatory activities can be planned and performed and sufficient health resources can be allocated to enable high-quality and timely public health interventions intended to mitigate the disease [42, 43]. In addition, once the disease finally appears, the authorities, medical professionals, and the entire society can immediately initiate the planned remediating activities, facilitating an effective and prompt intervention. Therefore, public health surveillance is a crucial system for the identification, prevention, and control of disease outbreaks [44] while enabling a better allocation of health resources [18].

### 2.1 Traditional system for surveillance

In the traditional system for public health surveillance, the responsible public health authorities continuously collect data on diseases, which are primarily derived from diagnosed cases that are reported by emergency departments, hospitals, laboratories, and other medical professionals [1, 45]. The identified illnesses are predominantly observed from clinical data such as diagnoses and clinical reports [45, 46]. It has, however, been argued that these passive surveillance strategies fail to provide complete and timely overviews of the diseases [47].

In addition, historical data are analyzed to identify and visualize disease-related trends, such as seasonal influenza, which has often been occurring around the same months throughout the preceding decades, and may, therefore, be predicted with a reasonable accuracy [6, 48]. In contrast, other researchers report that the influenza virus continuously evolves into slightly different variations each year, which makes forecasting the timing of influenza outbreaks as well as their impacts on the society very difficult [49]. However, the emergence of many other infectious diseases cannot be forecasted based on historical data [47, 50].

### 2.2 Limitations of the traditional system for surveillance

The methods that are commonly used in the traditional system for public health surveillance have been practiced for many decades. Although these systems are known to improve public health and reduce mortality, there is no consensus on the degree of usefulness of individual methods or on the best way to support their function [51]. Likewise, other literature has reported that the authorities have been unable to successfully reduce the incidence and prevalence of dengue and other mosquito-related epidemics [52].

Overall, these systems involve two significant limitations.

First, a major drawback is that these methods are inefficient and time-consuming [1, 20]. To identify confirmed cases, the system requires lab work that is very labor intensive to operate and maintain, which significantly increases the time required to process the clinical data [6]. For example, in the United States, the time required to collect and analyze the data about seasonal influenza and to produce and distribute the reports was estimated to be two weeks [49]. As a consequence, once these reports are finally distributed to politicians, medical professionals, and the general public, the reported findings are very likely to be outdated and may thus no longer accurately represent the current situation [53]. Therefore, these methods are not suited for the surveillance of novel infectious diseases such as SARS-CoV-2, which emerged in late 2019 [8] and involves an urgent need for real-time updates and demands immediate interventions [19]. While contact tracing is able to successfully trace infections, non-symptomatic and mild cases are nearly impossible to track and can easily enter other countries unnoticed.

Second, for communicable diseases such as malaria, disease trends can only be detected and analyzed after the actual outbreak of this disease [47, 50]. A severe limitation is that the outbreak and distribution of such diseases cannot be forecasted reliably [54].

Consequently, in the context of our highly dynamic society, the interconnectivity of all major cities by air travel leads to the very likely scenario that the outbreak of an infectious disease will easily spread around the globe in a matter of a few days, especially in non-symptomatic or mild cases [47, 55, 56].

### 2.3 Value of understanding text published to social media

Humans spend a significant amount of their time on social media communicating and disseminating information [4]. Social media platforms provide access to an abundance of valuable and public user-generated data that may be useful for public health surveillance and to detect, monitor, and prevent diseases [19, 45, 57, 58]. This makes social media platforms an important source for generating new knowledge [19].

A distinctive feature of social media is that it transforms its users into human sensors, although potentially biased and unreliable, who personally report on a variety of events and who may provide additional contextual information [6]. Furthermore, social media platforms often also collect geographical information about the precise locations of their users, which adds an additional and potentially valuable geographical dimension to these data [19, 59].

The analysis of textual content from social media is, however, not restricted to the field of diseases; an abundance of studies have used data from social media for application in many domains.

For example, user-generated content has been analyzed in a wide variety of domains, such as agriculture [60], business [61], and consumer behavior [62]; it has been used for purposes ranging from the detection of earthquakes [63], emergency and disaster management [64] to understanding migraines [65], presidential elections [66], political campaigns [67], and product design [68], to predicting the revenue of movies [69], forecasting sports events [70], identifying the topical interests of users [71], identifying trending topics [72], and investigating voting patterns in elections [73].

### 2.4 Natural language processing

The unstructured nature of social media content, compared to structured data, demands much more preprocessing and processing before it can be analyzed [50]. Most data that are generated today have an unstructured format, e.g., text [74]. Only a small fraction of data has a structured format, which can then be analyzed directly with well-established techniques from data mining [74].

In the preceding years, extensive techniques for processing human language have been developed and refined, and the relevant domain that emerged has been named natural language processing (hereafter NLP) [75, 76]. NLP resembles the science of using computers to understand human language, while text mining provides the required methods and algorithms.

The purpose of text mining is to “discover novel information in a timely manner from large-scale text collections by developing high performance algorithms for sourcing and converting unstructured textual data to a machine understandable format and then filtering this according to the needs of its users” [75]. Therefore, text mining is used for the automatic discovery of patterns, relationships, and high-quality insights from textual data [77, 78].

Among others, the domain of text mining includes the following major techniques [79–81]:

- extraction of concepts, entities, and the relationships between them [82];
- clustering text based on a measurement of similarity [83, 84];
- predicting words or other lexical units (as part of a word processor or chatbot) [85, 86];
- summarizing text in documents [82];
- discovering associations between words and other tokens [87];
- classification of text into various categories [78, 88]; and
- assigning affective states to text (sentiment analysis) [82].

These techniques are used abundantly among both researchers and professionals [50].

Sentiment analysis is a popular technique that is frequently used in the domain of text mining. Sentiment analysis involves the identification of attitudes, emotions, and opinions that people have in relation to an entity, which is observed from expressed human language [89, 90]. The opportunity derived from using sentiment analysis on content from social media is that it may enable innovative applications [20]. For example, sentiment analysis enables the identification of content as either a fact or an opinion (also called subjectivity). In addition, for opinions, sentiment analysis can also identify polarity, namely, whether an opinion is positive, neutral, or negative.

Furthermore, because text mining can be used to extract structured data from unstructured content, techniques used in data mining can subsequently be applied to analyze these structured features further [50]. NLP was used in medicine and public health [81] for, among others, allergies [91–93], depression [94–96], to gauge public health concerns [97], marijuana and drug abuse [98–101], obesity [102, 103], suicide-related thoughts and conversations [104–106], and tobacco and e-cigarette use [107–110].

## 3 Methodology

This qualitative systematic review was guided by the Preferred Reporting Items for Systematic Reviews and Meta-Analyses (PRISMA) guidelines [111, 112] (see S2 Appendix). However, most of the reviewed papers did not contain controlled trials, comparable statistical analysis, or comparable methodologies, making it impossible to apply the entire PRISMA checklist to this review. Therefore, we only applied items on the checklist if they were applicable, and thus, our review does not conform completely to the guidelines.

The following search strategy and procedures for study selection and analysis were used. The study selection, quality assessment of the included studies, and thematic analysis were performed by one author (PP). However, the procedures and findings were discussed by all authors, and potential disagreements were resolved by consensus.

### 3.1 Information sources

This systematic review is based on literature that was indexed by four large databases, namely, the ACM Digital Library, IEEE Xplore, PubMed, and Web of Science. These databases were selected because of their relevance to this topic.

The ACM Digital Library and IEEE Xplore databases were searched for publications in the fields of computer science, data science, information management, and information technology. IEEE Xplore was also selected because much research on this topic is exclusively published at conferences instead of in peer-reviewed journals. The Institute of Electrical and Electronics Engineering (IEEE) hosts many of these relevant conferences. Furthermore, PubMed was included because of its focus on literature in the domain of medicine and healthcare, while Web of Science is a very broad database that indexes the literature from many relevant disciplines, such as public policy and the social sciences.

### 3.2 Search strategy

An optimized and broad search strategy was formulated for each of the four databases (see S1 Appendix). Overall, the search strategy consisted of two blocks with search terms related to natural language processing and public health monitoring. In addition, database-specific filters were applied to narrow the search results further.

The first block, natural language processing, contained the search terms artificial intelligence, machine learning, text mining, computational linguistics, natural language processing, sentiment analysis, word embeddings, and Natural Language Toolkit. Abbreviations and wildcards were included to find alternative phrasing of these concepts. The OR operator was used to combine these search terms.

The second block, public health monitoring, contained the search terms public health surveillance, public health monitoring, and health monitoring. Experimental searches have indicated that these broader search terms resulted in the most relevant results. The OR operator was used to combine these search terms.

If supported by the database, subject headings such as MeSH terms for PubMed were also included in the search strategy. Subsequently, the AND operator was used to combine the queries from each block into the final search query.

The literature search was performed in March 2020. After executing the formulated search queries in each database, additional filters were manually applied to narrow the search results further. Although the precise filters were different across the databases, two examples of such filters are that publications were only written in the English language and that these studies were published in journals or presented at conferences.

For each of the four databases, all search results were then exported and subsequently imported into the same EndNote Library. Because these databases partially returned the same results, the deduplication strategy by Bramer et al. [113] was used to eliminate these duplicate publications from the EndNote Library. Consequently, the EndNote Library contained only unique results.

### 3.3 Process of study selection

The remaining publications were screened and selected using three subsequent phases based on their title, abstract, and full text. To avoid erroneously excluding publications, the screening in these phases was performed with high flexibility. Therefore, if there was any doubt concerning a publication’s eligibility or when insufficient information was provided to confidently exclude a manuscript, that publication was retained for further screening in a subsequent phase.

In the first phase, the titles of these publications were screened for their relevance to the topic of this systematic review. The titles of eligible studies indicated the analysis of textual content for the surveillance or monitoring of diseases.

In the second phase, the abstract and keywords of the remaining studies were screened for information indicating the analysis of textual content that was generated by users and published to at least social media, with the purpose of public health surveillance and monitoring of communicable diseases. As a result, studies that only analyzed news articles were considered irrelevant and were eliminated.

Finally, the third phase involved rigorous screening of the full text of the remaining publications. Eligible studies reported original and empirical research analyzing the textual content that the general public published to at least social media, with the purpose of surveilling and monitoring public health with respect to communicable diseases. This phase did not discriminate between geographies, social media platforms, or communicable diseases. However, publications that only investigated non-communicable diseases were eliminated. When studies investigated communicable diseases, this systematic review did not discriminate between the type of disease, i.e., all communicable diseases were included in this systematic review.

This resulted in a remaining subset of the identified publications that was included for further selection in this systematic review.

### 3.4 Selection criteria

Overall, eligible publications reported original and empirical research that reported findings on the application of analyzing user-generated textual content from social media for the monitoring and prediction of communicable diseases. Reviews, discussion papers, editorials, and papers that only proposed a framework for the analysis of social media content without the actual application and reporting of these findings were eliminated. All peer-reviewed journal articles and publications related to conferences were included.

In addition, although studies were considered relevant if they included textual content that was published to at least social media, this systematic review did not discriminate between the different social media platforms. All social media platforms were considered relevant and were included. Likewise, this systematic review included all papers irrespective of the language of the social media content used, the geography of these users and their content, or the authors of the identified publications.

This study aimed to aggregate the reported findings on the surveillance and monitoring of public health based on the experiences of the population that were published on social media. Therefore, papers were excluded if they only included content that was published on social media by authors other than the general public, such as governments, health professionals, and commercial entities.

### 3.5 Data analysis

In accordance with Kampmeijer et al. [114] and utilizing the process described by Pilipiec et al. [81], the included studies were first assessed according to their quality, which was operationalized as reliability and validity. A reliable study provided a thorough and complete description of the methods that were used for the data collection and data analysis, and this process was also considered repeatable [114]. A valid study reported results that were consistent with the research objective and the utilized research methods [114]. An ordinal scale was used to grade studies with respect to their reliability and validity as either low, medium, or high. Regardless of the quality level, all studies were included in this systematic review.

Directed qualitative content analysis, also called thematic analysis, was used to analyze the included studies [115]. Thematic analysis is a primary method for qualitative research that is widely used among qualitative researchers [116, 117]. Its popularity may be explained because thematic analysis is a highly flexible method that can produce trustworthy insights [116, 118].

The themes of interest were based on the objective of this systematic review. The following themes were extracted from these publications: authors, year of publication, publication type, name of communicable disease, social media platform used, sample size, language of the data, period of data collection, horizon of data collection, country, software used for natural language processing, methods and techniques used for natural language processing, investigated target, algorithm used for predicting the target, reported result, description of the results, reliability, and validity.

The extracted information from all included publications was used to create an extraction matrix. The results were summarized using tables, and a synthesis of this information was presented narratively. In addition to assessing the quality of the studies that were included in this systematic review, the PRISMA checklist [111, 112] in S2 Appendix was used to assess the quality of this systematic review.

## 4 Results

**Fig 1:**
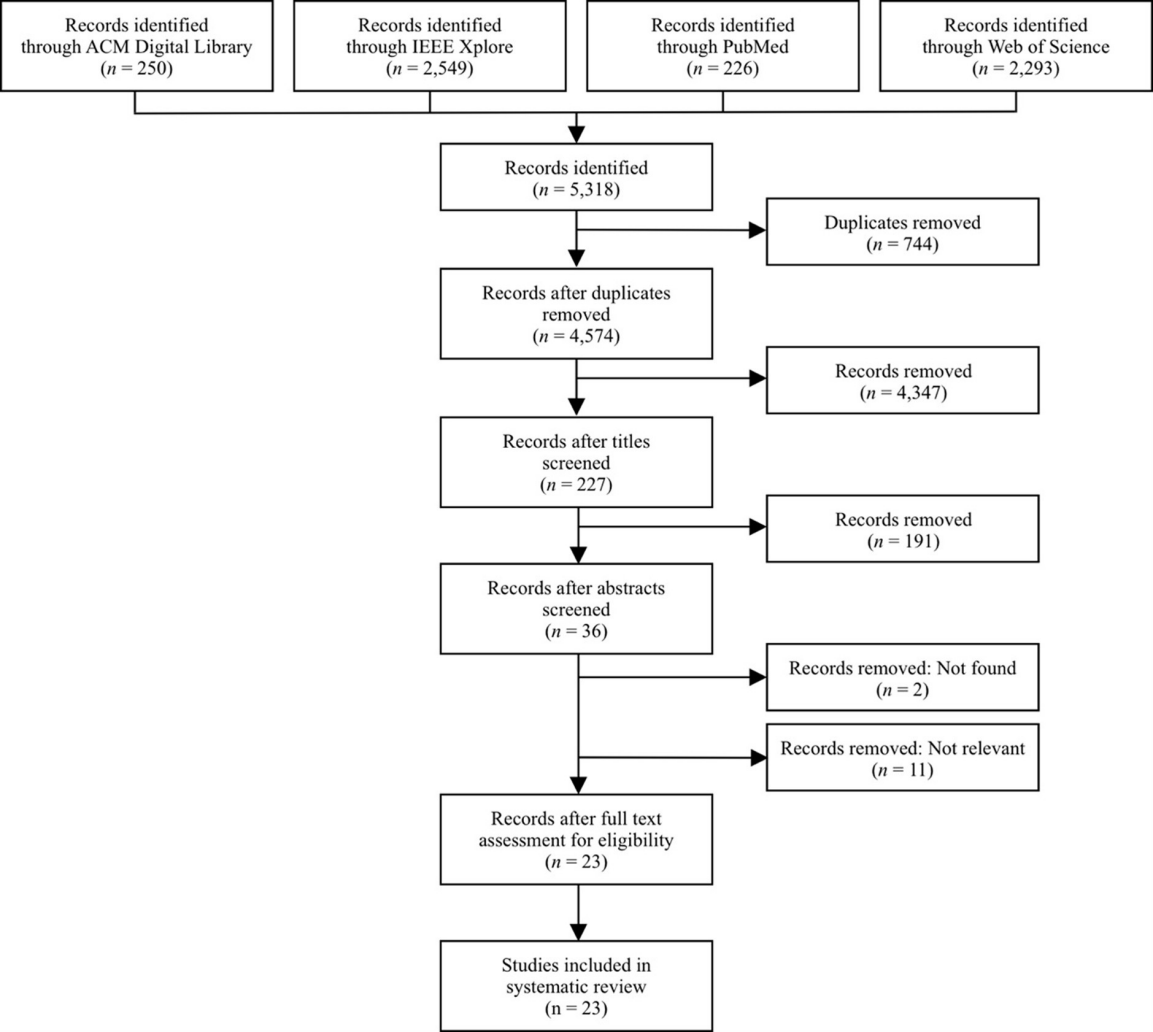
Results of study selection.

The flow diagram in Fig 1 presents the results of the studies that were selected to be included in this systematic review. The execution of the optimized search queries in the four databases (see S1 Appendix) yielded a total of 5,318 hits. Of these results, 250 records were identified through the ACM Digital Library, 2,549 records were found in IEEE Xplore, PubMed yielded 226 records, and Web of Science returned 2,293 records. However, 744 records were identified as duplicates and were, therefore, removed. This resulted in an EndNote Library with 4,574 unique records.

Subsequently, screening was performed in three consecutive phases to exclude irrelevant records, according to the process described in Sections 3.3 and 3.4.

In the first phase, 4,347 records were removed after screening the title, resulting in 227 remaining records. In the second phase, the records were screened based on their abstracts and keywords. The 191 records that were considered irrelevant were eliminated. This resulted in 36 remaining studies. In the third phase, the full texts of the records were screened. However, the full texts of two records could not be retrieved, and these studies were subsequently removed. Of these records, 11 records were considered not to be relevant and were excluded. This resulted in the identification of 23 eligible publications that were included in this systematic review. A detailed description of the characteristics of these studies is presented in S3 Appendix.

### 4.1 Study characteristics

Table 1 presents an extensive description of the studies that were included in this systematic review. All studies were published between 2010 and 2019. A majority of these studies (65.2%) were published in the last five years [6, 30, 44, 45, 53, 58, 74, 76, 119–125]. Most studies were published in 2015 (17.4%) [119–122] and 2016 (17.4%) [58, 123–125], while no studies were published in 2012.

**Table 1:** Description of studies analyzed (23 studies included).

Category	Sub-category	*n* (%)	Publications
Year	2010	2 (8.7)	[126, 128]
	2011	1 (4.4)	[127]
	2012	0 (0.0)	-
	2013	2 (8.7)	[18, 24]
	2014	3 (13.0)	[4, 28, 49]
	2015	4 (17.4)	[119–122]
	2016	4 (17.4)	[58, 123–125]
	2017	2 (8.7)	[44, 53]
	2018	2 (8.7)	[30, 45]
	2019	3 (13.0)	[6, 74, 76]
Publication type	Conference proceeding	8 (34.8)	[4, 18, 24, 76, 121, 124, 127, 128]
	Journal article	15 (65.2)	[6, 28, 30, 44, 45, 49, 53, 58, 74, 119, 120, 122, 123, 125, 126]
Communicable disease	Dengue	3 (11.5)	[76, 125, 127]
	Ebola	1 (3.9)	[120]
	HIV/AIDS	1 (3.9)	[128]
	Influenza	16 (61.5)	[4, 6, 18, 24, 28, 30, 44, 45, 49, 53, 58, 121–124, 126]
	Listeria	1 (3.9)	[24]
	Measles	3 (11.5)	[24, 74, 119]
	Tuberculosis	1 (3.9)	[24]
Social media platform	Sina Weibo	2 (8.7)	[4, 122]
	Twitter	20 (87.0)	[6, 18, 24, 28, 30, 44, 45, 49, 53, 58, 74, 76, 119–121, 123–127]
	Yahoo! Knowledge	1 (4.4)	[128]
Sample size	Less than 25,000	8 (34.8)	[28, 45, 74, 76, 119, 124, 125, 128]
	25,000 to 99,999	3 (13.0)	[44, 120, 121]
	100,000 to 249,999	1 (4.4)	[49]
	250,000 to 999,999	2 (8.7)	[122, 127]
	1,000,000 or more	7 (30.4)	[4, 6, 18, 30, 53, 123, 126]
	Unknown	2 (8.7)	[24, 58]
Language of data	Arabic	1 (4.2)	[6]
	English	5 (20.8)	[6, 44, 120, 124, 126]
	Japanese	1 (4.2)	[30]
	Mandarin	2 (8.3)	[4, 122]
	Unknown	15 (62.5)	[18, 24, 28, 45, 49, 53, 58, 74, 76, 119, 121, 123, 125, 127, 128]
Horizon of data collection	Less than 1 month	2 (8.7)	[74, 120]
	1 to 6 months	9 (39.1)	[4, 6, 18, 24, 28, 44, 49, 122, 124]
	7 to 12 months	3 (13.0)	[119, 126, 127]
	13 to 18 months	1 (4.4)	[121]
	19 to 24 months	1 (4.4)	[123]
	25 or more months	5 (21.7)	[30, 53, 76, 125, 128]
	Unknown	2 (8.7)	[45, 58]
Country	Australia	1 (4.2)	[121]
	Brazil	3 (12.5)	[76, 125, 127]
	Canada	1 (4.2)	[124]
	China	2 (8.3)	[4, 122]
	India	1 (4.2)	[45]
	Japan	1 (4.2)	[30]
	New Zealand	1 (4.2)	[121]
	Taiwan	1 (4.2)	[128]
	The Netherlands	1 (4.2)	[119]
	United Arab Emirates	1 (4.2)	[6]
	United States	6 (25.0)	[24, 28, 44, 49, 53, 58]
	Unknown	5 (20.8)	[18, 74, 120, 123, 126]
Software for NLP	Apache Lucene’s PorterStemFilter	1 (3.7)	[124]
	Apache Lucene’s StopFilter	1 (3.7)	[124]
	Datasift service	1 (3.7)	[123]
	Natural Language Toolkit	1 (3.7)	[6]
	OpenNLP	1 (3.7)	[124]
	Stanford CoreNLP	2 (7.4)	[44, 124]
	The Stanford parser	1 (3.7)	[44]
	Unknown	19 (70.4)	[4, 18, 24, 28, 30, 45, 49, 53, 58, 74, 76, 119–122, 125–128]
Processing for NLP	Content analysis	1 (1.7)	[120]
	Detecting URLs	1 (1.7)	[124]
	Dimensionality reduction	1 (1.7)	[45]
	Feature weighting	1 (1.7)	[45]
	Homogenization	2 (3.3)	[74, 124]
	Language categorization	1 (1.7)	[6]
	LDA topics	1 (1.7)	[53]
	Lemmatization	3 (5.0)	[44, 45, 123]
	*n*-gram generation	6 (10.0)	[24, 44, 53, 74, 76, 120]
	Normalization using frequency-based methods	1 (1.7)	[45]
	Remove symbols and URLs	1 (1.7)	[120]
	Sentiment analysis	5 (8.3)	[24, 119, 124, 126, 127]
	Stemming	7 (11.7)	[6, 24, 45, 74, 76, 123, 124]
	Stop word removal	6 (10.0)	[6, 18, 45, 74, 76, 124]
	Term filtering	1 (1.7)	[74]
	Term Frequency—Inverse Document Frequency (TF-IDF)	8 (13.3)	[4, 24, 44, 49, 53, 58, 74, 76]
	Text embeddings	1 (1.7)	[53]
	Thematic analysis	1 (1.7)	[119]
	Topic detection	1 (1.7)	[120]
	Tokenization	4 (6.7)	[6, 18, 45, 74]
	Tweet filtering	1 (1.7)	[6]
	Unknown	6 (10.0)	[28, 30, 121, 122, 125, 128]
Algorithm for prediction of target	1-gram Term Frequency classifier	1 (2.4)	[44]
	Association rule mapping	1 (2.4)	[127]
	Chi-Square test	1 (2.4)	[126]
	Correlation analysis	2 (4.9)	[53, 119]
	Decision Tree	2 (4.9)	[45, 76]
	Fuzzy Algorithm for Extraction, Monitoring and Classification of infectious Diseases (FAEMC-ID)	1 (2.4)	[74]
	Hidden Markov Model	1 (2.4)	[123]
	*k*-Means clustering	2 (4.9)	[4, 120]
	*k*-Nearest Neighbors	1 (2.4)	[4]
	Latent Dirichlet allocation (LDA)	1 (2.4)	[44]
	Linear regression	5 (12.2)	[6, 28, 53, 125, 127]
	Maximum entropy	1 (2.4)	[125]
	Naïve Bayes	5 (12.2)	[24, 45, 76, 124, 125]
	Random Forest	1 (2.4)	[45]
	Recurrent neural networks with Long short-term memory (LSTM)	1 (2.4)	[53]
	ST-DBSCAN	1 (2.4)	[127]
	Support vector machines	10 (24.4)	[4, 24, 30, 44, 45, 49, 53, 58, 125, 128]
	Time series	1 (2.4)	[120]
	Unknown	3 (7.3)	[18, 121, 122]
Result	Negative	0 (0.0)	-
	Positive	23 (100.0)	[4, 6, 18, 24, 28, 30, 44, 45, 49, 53, 58, 74, 76, 119–128]
Reliability	Low	3 (13.0)	[18, 122, 128]
	Medium	16 (69.6)	[4, 24, 28, 30, 45, 49, 53, 58, 74, 76, 120, 121, 124–127]
	High	4 (17.4)	[6, 44, 119, 123]
Validity	Low	3 (13.0)	[18, 122, 128]
	Medium	16 (69.6)	[4, 24, 28, 30, 45, 49, 53, 58, 74, 76, 120, 121, 124–127]
	High	4 (17.4)	[6, 44, 119, 123]

A majority of the studies (65.2%) were published as a peer-reviewed journal article [6, 28, 30, 44, 45, 49, 53, 58, 74, 119, 120, 122, 123, 125, 126], while the remaining 34.8 percent of the studies were published at a conference [4, 18, 24, 76, 121, 124, 127, 128].

The included studies investigated a total of seven communicable diseases, and publications may have reported findings on multiple diseases. Influenza was studied most frequently (61.5%) [4, 6, 18, 24, 28, 30, 44, 45, 49, 53, 58, 121–124, 126]. This was followed by dengue [76, 125, 127] and measles [24, 74, 119], which were each studied in 11.5 percent of the included studies. Ebola [120], HIV/AIDS [128], listeria [24], and tuberculosis [24] were studied least often (3.9% each). Only one study investigated more than one communicable disease; this study analyzed four diseases (i.e., influenza, listeria, measles, and tuberculosis) [24].

The results in Table 1 for the input sources, employed methods, and study effectiveness are discussed in the subsequent subsections.

### 4.2 Input sources

User-generated textual content was retrieved from three social media platforms (see Table 1). Content published to Twitter was used most frequently (87.0%) [6, 18, 24, 28, 30, 44, 45, 49, 53, 58, 74, 76, 119–121, 123–127]. The platforms Sina Weibo (8.7%) [4, 122] and Yahoo! Knowledge (4.4%) [128] were studied the least. All studies only included content from one social media platform.

There was a vast difference in the sample size that was included in the studies. This sample size ranged from 667 tweets [76] to 171,027,275 tweets [53]. Overall, in most studies, the sample size was either less than 25,000 (34.8%) [28, 45, 74, 76, 119, 124, 125, 128] or one million or more (30.4%) [4, 6, 18, 30, 53, 123, 126]. In 26.1 percent of the studies, the sample size was between 25,000 and 999,999 items [44, 49, 120–122, 127]. However, 8.7 percent of the studies failed to report the sample size [24, 58].

The studies investigated user-generated textual content that was written in different languages. The content was most often written in English (20.8%) [6, 44, 120, 124, 126]. Content written in Mandarin (8.3%) [4, 122], Arabic (4.2%) [6], and Japanese (4.2%) [30] was included less frequently. Only one study (4.4%) investigated content that was written in more than one language, namely, in Arabic and English [6]. However, a vast majority of the studies (65.2%) failed to report the language of the content that was analyzed [18, 24, 28, 45, 49, 53, 58, 74, 76, 119, 121, 123, 125, 127, 128].

The time horizon with respect to the date of publication of the analyzed data was also diverse, ranging from one week [74, 120] to 106 months [76]. However, most of the studies (39.1%) analyzed samples that were published in periods ranging from one to six months [4, 6, 18, 24, 28, 44, 49, 122, 124]. Additionally, more than one-fifth of the studies (21.7%) analyzed content that was published during a period of at least 25 months [30, 53, 76, 125, 128]. Only two studies (8.7%) included content that was published during a period less than one month [74, 120]. However, two studies (8.7%) did not disclose the precise time horizon for the publication dates of the included samples [45, 58].

The included studies analyzed content related to 11 countries. Posts published in the United States were analyzed most often (25.0%) [24, 28, 44, 49, 53, 58], followed by those published in Brazil (12.5%) [76, 125, 127] and China (8.3%) [4, 122]. The remaining countries are Australia [121], Canada [124], India [45], Japan [30], New Zealand [121], Taiwan [128], the Netherlands [119], and the United Arab Emirates [6], which each were studied in 4.2 percent of the included studies. Five studies (21.7%), however, failed to disclose the geographical locations of the included posts [18, 74, 120, 123, 126]. With the exception of Robinson et al. [121], who analyzed posts from Australia and New Zealand, the remaining studies included content from only one country.

### 4.3 Employed methods

In the forthcoming synthesis of the methods that publications employed, the reader should be aware that our objective was to investigate the methods that authors utilized and explicitly mentioned in their manuscript. We acknowledge the possibility that authors applied common methods for text analysis, such as stop word removal, tokenization, stemming, and lemmatization, but failed to report this in their manuscript. This may be explained by the fact that these preprocessing methods are highly common in natural language processing.

Although all studies analyzed textual content using some variant of natural language processing, a majority of the studies (82.6%) failed to disclose information on the software that was used [4, 18, 24, 28, 30, 45, 49, 53, 58, 74, 76, 119–122, 125–128] (see Table 1). Only four studies (17.4%) provided information about the utilized software [6, 44, 123, 124]. When studies reported the software utilized, seven software packages were discussed. Stanford CoreNLP was used most often (7.4%) [44, 124], while Apache Lucene’s PorterStemFilter [124], Apache Lucene’s StopFilter [124], Datasift service [123], Natural Language Toolkit [6], OpenNLP [124], and The Stanford parser [44] were used the least (3.7% each). Additionally, studies could utilize more than one software package. Byrd et al. [124] used four software packages for natural language processing.

The studies reported 21 methods and algorithms for natural language preprocessing. Term Frequency—Inverse Document Frequency (TF-IDF) was used most often (13.3%) [4, 24, 44, 49, 53, 58, 74, 76]. This was followed by stemming (11.7%) [6, 24, 45, 74, 76, 123, 124], *n*-gram generation (10.0%) [24, 44, 53, 74, 76, 120], stop word removal (10.0%) [6, 18, 45, 74, 76, 124], sentiment analysis (8.3%) [24, 119, 124, 126, 127], tokenization (6.7%) [6, 18, 45, 74], and lemmatization (5.0%) [44, 45, 123]. The remaining 14 methods and algorithms were used in 25.0 percent of the studies. Although the majority of studies reported detailed information about these methods and algorithms, more than a quarter (26.1%) of the included studies did not disclose this information [28, 30, 121, 122, 125, 128].

A vast majority of the studies (87.0%) reported information on the algorithms that were used to predict the target, i.e., the outcome estimated using the textual content. A total of 18 algorithms were utilized. Support vector machines (24.4%) [4, 24, 30, 44, 45, 49, 53, 58, 125, 128], linear regression (12.2%) [6, 28, 53, 125, 127], and Naïve Bayes (12.2%) [24, 45, 76, 124, 125] were used most often. These three supervised learning algorithms are highly popular among data mining practitioners. Therefore, their utilization was expected for the prediction of a numerical outcome or a category.

Although a vast majority of studies disclosed information on the algorithm used, 13.0 percent of the studies [18, 121, 122] did not provide such information.

### 4.4 Study effectiveness

All studies reported positive results on using user-generated textual content from social media to monitor or surveille communicable diseases (see Table 1). Although positive findings were reported, it was explicitly discussed in one study that lower educated males of older age are less likely to disclose information on the infectious disease dengue to Twitter, making this platform less suitable for the monitoring and surveillance of this disease among this group of persons [125].

Furthermore, the quality of the included studies was evaluated based on its reliability and validity. A majority of studies [4, 24, 28, 30, 45, 49, 53, 58, 74, 76, 120, 121, 124–127] had medium reliability (69.6%) and validity (69.6%). Four studies [6, 44, 119, 123] were found to have high reliability (17.4%) and validity (17.4%). This means that these studies not only provided a complete description of the methods that were used for the data collection and data analysis and that this process was considered repeatable, but these studies also reported results that are consistent with the research objective and the utilized research methods [114]. For the remaining studies [18, 122, 128], the reliability (13.0%) and validity (13.0%) were, however, low.

### 4.5 Analysis of publications by publication type

Furthermore, the included publications were additionally analyzed based on the publication type, i.e., conference proceedings and journal articles (see Table 2). Of these publications, eight studies (34.8%) were presented at a conference [4, 18, 24, 76, 121, 124, 127, 128], and 15 studies (65.2%) were published in a peer-reviewed journal [6, 28, 30, 44, 45, 49, 53, 58, 74, 119, 120, 122, 123, 125, 126]. Overall, the analysis indicates that both conference proceedings and journal articles reported comparable findings.

**Table 2:** Description of studies analyzed by publication type (23 studies included).

Category	Sub-category	Conference proceeding *n* (%)	Journal article *n* (%)	Publications
Year	2010	1 (4.4)	1 (4.6)	[126, 128]
	2011	1 (4.4)	0 (0.0)	[127]
	2012	0 (0.0)	0 (0.0)	-
	2013	2 (8.7)	0 (0.0)	[18, 24]
	2014	1 (4.4)	2 (8.7)	[4, 28, 49]
	2015	1 (4.4)	3 (13.0)	[119–122]
	2016	1 (4.4)	3 (13.0)	[58, 123–125]
	2017	0 (0.0)	2 (8.7)	[44, 53]
	2018	0 (0.0)	2 (8.7)	[30, 45]
	2019	1 (4.4)	2 (8.7)	[6, 74, 76]
Communicable disease	Dengue	2 (7.7)	1 (3.9)	[76, 125, 127]
	Ebola	0 (0.0)	1 (3.9)	[120]
	HIV/AIDS	1 (3.9)	0 (0.0)	[128]
	Influenza	5 (19.2)	11 (42.3)	[4, 6, 18, 24, 28, 30, 44, 45, 49, 53, 58, 121–124, 126]
	Listeria	1 (3.9)	0 (0.0)	[24]
	Measles	1 (3.9)	2 (7.7)	[24, 74, 119]
	Tuberculosis	1 (3.9)	0 (0.0)	[24]
Social media platform	Sina Weibo	1 (4.4)	1 (4.4)	[4, 122]
	Twitter	6 (26.1)	14 (60.9)	[6, 18, 24, 28, 30, 44, 45, 49, 53, 58, 74, 76, 119–121, 123–127]
	Yahoo! Knowledge	1 (4.4)	0 (0.0)	[128]
Sample size	Less than 25,000	3 (13.0)	5 (21.7)	[28, 45, 74, 76, 119, 124, 125, 128]
	25,000 to 99,999	1 (4.4)	2 (8.7)	[44, 120, 121]
	100,000 to 249,999	0 (0.0)	1 (4.4)	[49]
	250,000 to 999,999	1 (4.4)	1 (4.4)	[122, 127]
	1,000,000 or more	2 (8.7)	5 (21.7)	[4, 6, 18, 30, 53, 123, 126]
	Unknown	1 (4.4)	1 (4.4)	[24, 58]
Language of data	Arabic	0 (0.0)	1 (4.2)	[6]
	English	1 (4.2)	4 (16.7)	[6, 44, 120, 124, 126]
	Japanese	0 (0.0)	1 (4.2)	[30]
	Mandarin	1 (4.2)	1 (4.2)	[4, 122]
	Unknown	6 (25.0)	9 (37.5)	[18, 24, 28, 45, 49, 53, 58, 74, 76, 119, 121, 123, 125, 127, 128]
Horizon of data collection	Less than 1 month	0 (0.0)	2 (8.7)	[74, 120]
	1 to 6 months	4 (17.4)	5 (21.7)	[4, 6, 18, 24, 28, 44, 49, 122, 124]
	7 to 12 months	1 (4.4)	2 (8.7)	[119, 126, 127]
	13 to 18 months	1 (4.4)	0 (0.0)	[121]
	19 to 24 months	0 (0.0)	1 (4.4)	[123]
	25 or more months	2 (8.7)	3 (13.0)	[30, 53, 76, 125, 128]
	Unknown	0 (0.0)	2 (8.7)	[45, 58]
Country	Australia	1 (4.2)	0 (0.0)	[121]
	Brazil	2 (8.3)	1 (4.2)	[76, 125, 127]
	Canada	1 (4.2)	0 (0.0)	[124]
	China	1 (4.2)	1 (4.2)	[4, 122]
	India	0 (0.0)	1 (4.2)	[45]
	Japan	0 (0.0)	1 (4.2)	[30]
	New Zealand	1 (4.2)	0 (0.0)	[121]
	Taiwan	1 (4.2)	0 (0.0)	[128]
	The Netherlands	0 (0.0)	1 (4.2)	[119]
	United Arab Emirates	0 (0.0)	1 (4.2)	[6]
	United States	1 (4.2)	5 (20.8)	[24, 28, 44, 49, 53, 58]
	Unknown	1 (4.2)	4 (16.7)	[18, 74, 120, 123, 126]
Software for NLP	Apache Lucene’s PorterStemFilter	1 (3.7)	0 (0.0)	[124]
	Apache Lucene’s StopFilter	1 (3.7)	0 (0.0)	[124]
	Datasift service	0 (0.0)	1 (3.7)	[123]
	Natural Language Toolkit	0 (0.0)	1 (3.7)	[6]
	OpenNLP	1 (3.7)	0 (0.0)	[124]
	Stanford CoreNLP	1 (3.7)	1 (3.7)	[44, 124]
	The Stanford parser	0 (0.0)	1 (3.7)	[44]
	Unknown	7 (25.9)	12 (44.4)	[4, 18, 24, 28, 30, 45, 49, 53, 58, 74, 76, 119–122, 125–128]
Processing for NLP	Content analysis	0 (0.0)	1 (1.7)	[120]
	Detecting URLs	1 (1.7)	0 (0.0)	[124]
	Dimensionality reduction	0 (0.0)	1 (1.7)	[45]
	Feature weighting	0 (0.0)	1 (1.7)	[45]
	Homogenization	1 (1.7)	1 (1.7)	[74, 124]
	Language categorization	0 (0.0)	1 (1.7)	[6]
	LDA topics	0 (0.0)	1 (1.7)	[53]
	Lemmatization	0 (0.0)	3 (5.0)	[44, 45, 123]
	*n*-gram generation	2 (3.3)	4 (6.7)	[24, 44, 53, 74, 76, 120]
	Normalization using frequency-based methods	0 (0.0)	1 (1.7)	[45]
	Remove symbols and URLs	0 (0.0)	1 (1.7)	[120]
	Sentiment analysis	3 (5.0)	2 (3.3)	[24, 119, 124, 126, 127]
	Stemming	3 (5.0)	4 (6.7)	[6, 24, 45, 74, 76, 123, 124]
	Stop word removal	3 (5.0)	3 (5.0)	[6, 18, 45, 74, 76, 124]
	Term filtering	0 (0.0)	1 (1.7)	[74]
	Term Frequency—Inverse Document Frequency (TF-IDF)	3 (5.0)	5 (8.3)	[4, 24, 44, 49, 53, 58, 74, 76]
	Text embeddings	0 (0.0)	1 (1.7)	[53]
	Thematic analysis	0 (0.0)	1 (1.7)	[119]
	Topic detection	0 (0.0)	1 (1.7)	[120]
	Tokenization	1 (1.7)	3 (5.0)	[6, 18, 45, 74]
	Tweet filtering	0 (0.0)	1 (1.7)	[6]
	Unknown	2 (3.3)	4 (6.7)	[28, 30, 121, 122, 125, 128]
Algorithm for prediction of target	1-gram Term Frequency classifier	0 (0.0)	1 (2.4)	[44]
	Association rule mapping	1 (2.4)	0 (0.0)	[127]
	Chi-Square test	0 (0.0)	1 (2.4)	[126]
	Correlation analysis	0 (0.0)	2 (4.9)	[53, 119]
	Decision Tree	1 (2.4)	1 (2.4)	[45, 76]
	Fuzzy Algorithm for Extraction, Monitoring and Classification of infectious Diseases (FAEMC-ID)	0 (0.0)	1 (2.4)	[74]
	Hidden Markov Model	0 (0.0)	1 (2.4)	[123]
	*k*-Means clustering	1 (2.4)	1 (2.4)	[4, 120]
	*k*-Nearest Neighbors	1 (2.4)	0 (0.0)	[4]
	Latent Dirichlet allocation (LDA)	0 (0.0)	1 (2.4)	[44]
	Linear regression	1 (2.4)	4 (9.8)	[6, 28, 53, 125, 127]
	Maximum entropy	0 (0.0)	1 (2.4)	[125]
	Naïve Bayes	3 (7.3)	2 (4.9)	[24, 45, 76, 124, 125]
	Random Forest	0 (0.0)	1 (2.4)	[45]
	Recurrent neural networks with Long short-term memory (LSTM)	0 (0.0)	1 (2.4)	[53]
	ST-DBSCAN	1 (2.4)	0 (0.0)	[127]
	Support vector machines	3 (7.3)	7 (17.1)	[4, 24, 30, 44, 45, 49, 53, 58, 125, 128]
	Time series	0 (0.0)	1 (2.4)	[120]
	Unknown	2 (4.9)	1 (2.4)	[18, 121, 122]
Result	Negative	0 (0.0)	0 (0.0)	-
	Positive	8 (34.8)	15 (65.2)	[4, 6, 18, 24, 28, 30, 44, 45, 49, 53, 58, 74, 76, 119–128]
Reliability	Low	2 (8.7)	1 (4.4)	[18, 122, 128]
	Medium	6 (26.1)	10 (43.5)	[4, 24, 28, 30, 45, 49, 53, 58, 74, 76, 120, 121, 124–127]
	High	0 (0.0)	4 (17.4)	[6, 44, 119, 123]
Validity	Low	2 (8.7)	1 (4.4)	[18, 122, 128]
	Medium	6 (26.1)	10 (43.5)	[4, 24, 28, 30, 45, 49, 53, 58, 74, 76, 120, 121, 124–127]
	High	0 (0.0)	4 (17.4)	[6, 44, 119, 123]

However, there are a few notable and novel differences regarding the following themes: the type of communicable disease, social media platform, geographical locations of included samples, and the quality of these studies, which was operationalized as reliability and validity.

There were no notable differences between the studies with respect to the communicable diseases that were investigated. In both conference proceedings and journal articles, there was a strong emphasis on monitoring and surveilling influenza [4, 6, 18, 24, 28, 30, 44, 45, 49, 53, 58, 121–124, 126]. However, several diseases were only investigated in journal articles (i.e., Ebola [120]), while HIV/AIDS [128], listeria [24], and tuberculosis [24] were only investigated in conference proceedings.

In addition, both conference proceedings and journal articles placed a strong emphasis on the social media platform Twitter [6, 18, 24, 28, 30, 44, 45, 49, 53, 58, 74, 76, 119–121, 123–127]. Sina Weibo [4, 122], however, received the least attention in both types of publications. Additionally, Yahoo! Knowledge [128] was only studied in one conference proceeding but not in a journal article.

Although both conference proceedings and journal articles investigated content that was published in various countries, journal articles relatively more often included textual content that was published in the United States [28, 44, 49, 53, 58]. However, journal articles were also more likely to lack a disclosure of geographical information [74, 120, 123, 126]. There were, however, no notable differences between the continents.

Last, only journal articles were evaluated as having high reliability and high validity [6, 44, 119, 123]. No conference proceedings were assessed as high on these themes. In contrast, conference proceedings were more likely to have low reliability and low validity [18, 128] relative to journal articles [122]. There were no notable differences between conference proceedings and journal articles that were assessed as having a medium quality. Overall, journal articles, therefore, had a higher quality than conference proceedings.

### 4.6 Analysis of publications by social media platform

In addition to the analyses above, the included publications were also analyzed based on the social media platforms from which the content was extracted (see Table 3). These social media platforms are Sina Weibo, Twitter, and Yahoo! Knowledge. Overall, comparable findings were reported across the groups of the literature that utilized content from each of the social media platforms.

**Table 3:** Description of studies analyzed by social media platform (23 studies included).

Category	Sub-category	Sina Weibo *n* (%)	Twitter *n* (%)	Yahoo! Knowledge *n* (%)	Publications
Year	2010	0 (0.0)	1 (4.4)	1 (4.4)	[126, 128]
	2011	0 (0.0)	1 (4.4)	0 (0.0)	[127]
	2012	0 (0.0)	0 (0.0)	0 (0.0)	-
	2013	0 (0.0)	2 (8.7)	0 (0.0)	[18, 24]
	2014	1 (4.4)	2 (8.7)	0 (0.0)	[4, 28, 49]
	2015	1 (4.4)	3 (13.0)	0 (0.0)	[119–122]
	2016	0 (0.0)	4 (17.4)	0 (0.0)	[58, 123–125]
	2017	0 (0.0)	2 (8.7)	0 (0.0)	[44, 53]
	2018	0 (0.0)	2 (8.7)	0 (0.0)	[30, 45]
	2019	0 (0.0)	3 (13.0)	0 (0.0)	[6, 74, 76]
Publication type	Conference proceeding	1 (4.4)	6 (26.1)	1 (4.4)	[4, 18, 24, 76, 121, 124, 127, 128]
	Journal article	1 (4.4)	14 (60.9)	0 (0.0)	[6, 28, 30, 44, 45, 49, 53, 58, 74, 119, 120, 122, 123, 125, 126]
Communicable disease	Dengue	0 (0.0)	3 (11.5)	0 (0.0)	[76, 125, 127]
	Ebola	0 (0.0)	1 (3.9)	0 (0.0)	[120]
	HIV/AIDS	0 (0.0)	0 (0.0)	1 (3.9)	[128]
	Influenza	2 (7.7)	14 (53.9)	0 (0.0)	[4, 6, 18, 24, 28, 30, 44, 45, 49, 53, 58, 121–124, 126]
	Listeria	0 (0.0)	1 (3.9)	0 (0.0)	[24]
	Measles	0 (0.0)	3 (11.5)	0 (0.0)	[24, 74, 119]
	Tuberculosis	0 (0.0)	1 (3.9)	0 (0.0)	[24]
Sample size	Less than 25,000	0 (0.0)	7 (30.4)	1 (4.4)	[28, 45, 74, 76, 119, 124, 125, 128]
	25,000 to 99,999	0 (0.0)	3 (13.0)	0 (0.0)	[44, 120, 121]
	100,000 to 249,999	0 (0.0)	1 (4.4)	0 (0.0)	[49]
	250,000 to 999,999	1 (4.4)	1 (4.4)	0 (0.0)	[122, 127]
	1,000,000 or more	1 (4.4)	6 (26.1)	0 (0.0)	[4, 6, 18, 30, 53, 123, 126]
	Unknown	0 (0.0)	2 (8.7)	0 (0.0)	[24, 58]
Language of data	Arabic	0 (0.0)	1 (4.2)	0 (0.0)	[6]
	English	0 (0.0)	5 (20.8)	0 (0.0)	[6, 44, 120, 124, 126]
	Japanese	0 (0.0)	1 (4.2)	0 (0.0)	[30]
	Mandarin	2 (8.3)	0 (0.0)	0 (0.0)	[4, 122]
	Unknown	0 (0.0)	14 (58.3)	1 (4.2)	[18, 24, 28, 45, 49, 53, 58, 74, 76, 119, 121, 123, 125, 127, 128]
Horizon of data collection	Less than 1 month	0 (0.0)	2 (8.7)	0 (0.0)	[74, 120]
	1 to 6 months	2 (8.7)	7 (30.4)	0 (0.0)	[4, 6, 18, 24, 28, 44, 49, 122, 124]
	7 to 12 months	0 (0.0)	3 (13.0)	0 (0.0)	[119, 126, 127]
	13 to 18 months	0 (0.0)	1 (4.4)	0 (0.0)	[121]
	19 to 24 months	0 (0.0)	1 (4.4)	0 (0.0)	[123]
	25 or more months	0 (0.0)	4 (17.4)	1 (4.4)	[30, 53, 76, 125, 128]
	Unknown	0 (0.0)	2 (8.7)	0 (0.0)	[45, 58]
Country	Australia	0 (0.0)	1 (4.2)	0 (0.0)	[121]
	Brazil	0 (0.0)	3 (12.5)	0 (0.0)	[76, 125, 127]
	Canada	0 (0.0)	1 (4.2)	0 (0.0)	[124]
	China	2 (8.3)	0 (0.0)	0 (0.0)	[4, 122]
	India	0 (0.0)	1 (4.2)	0 (0.0)	[45]
	Japan	0 (0.0)	1 (4.2)	0 (0.0)	[30]
	New Zealand	0 (0.0)	1 (4.2)	0 (0.0)	[121]
	Taiwan	0 (0.0)	0 (0.0)	1 (4.2)	[128]
	The Netherlands	0 (0.0)	1 (4.2)	0 (0.0)	[119]
	United Arab Emirates	0 (0.0)	1 (4.2)	0 (0.0)	[6]
	United States	0 (0.0)	6 (25.0)	0 (0.0)	[24, 28, 44, 49, 53, 58]
	Unknown	0 (0.0)	5 (20.8)	0 (0.0)	[18, 74, 120, 123, 126]
Software for NLP	Apache Lucene’s PorterStemFilter	0 (0.0)	1 (3.7)	0 (0.0)	[124]
	Apache Lucene’s StopFilter	0 (0.0)	1 (3.7)	0 (0.0)	[124]
	Datasift service	0 (0.0)	1 (3.7)	0 (0.0)	[123]
	Natural Language Toolkit	0 (0.0)	1 (3.7)	0 (0.0)	[6]
	OpenNLP	0 (0.0)	1 (3.7)	0 (0.0)	[124]
	Stanford CoreNLP	0 (0.0)	2 (7.4)	0 (0.0)	[44, 124]
	The Stanford parser	0 (0.0)	1 (3.7)	0 (0.0)	[44]
	Unknown	2 (7.4)	16 (59.3)	1 (3.7)	[4, 18, 24, 28, 30, 45, 49, 53, 58, 74, 76, 119–122, 125–128]
Processing for NLP	Content analysis	0 (0.0)	1 (1.7)	0 (0.0)	[120]
	Detecting URLs	0 (0.0)	1 (1.7)	0 (0.0)	[124]
	Dimensionality reduction	0 (0.0)	1 (1.7)	0 (0.0)	[45]
	Feature weighting	0 (0.0)	1 (1.7)	0 (0.0)	[45]
	Homogenization	0 (0.0)	2 (3.3)	0 (0.0)	[74, 124]
	Language categorization	0 (0.0)	1 (1.7)	0 (0.0)	[6]
	LDA topics	0 (0.0)	1 (1.7)	0 (0.0)	[53]
	Lemmatization	0 (0.0)	3 (5.0)	0 (0.0)	[44, 45, 123]
	*n*-gram generation	0 (0.0)	6 (10.0)	0 (0.0)	[24, 44, 53, 74, 76, 120]
	Normalization using frequency-based methods	0 (0.0)	1 (1.7)	0 (0.0)	[45]
	Remove symbols and URLs	0 (0.0)	1 (1.7)	0 (0.0)	[120]
	Sentiment analysis	0 (0.0)	5 (8.3)	0 (0.0)	[24, 119, 124, 126, 127]
	Stemming	0 (0.0)	7 (11.7)	0 (0.0)	[6, 24, 45, 74, 76, 123, 124]
	Stop word removal	0 (0.0)	6 (10.0)	0 (0.0)	[6, 18, 45, 74, 76, 124]
	Term filtering	0 (0.0)	1 (1.7)	0 (0.0)	[74]
	Term Frequency—Inverse Document Frequency (TF-IDF)	1 (1.7)	7 (11.7)	0 (0.0)	[4, 24, 44, 49, 53, 58, 74, 76]
	Text embeddings	0 (0.0)	1 (1.7)	0 (0.0)	[53]
	Thematic analysis	0 (0.0)	1 (1.7)	0 (0.0)	[119]
	Topic detection	0 (0.0)	1 (1.7)	0 (0.0)	[120]
	Tokenization	0 (0.0)	4 (6.7)	0 (0.0)	[6, 18, 45, 74]
	Tweet filtering	0 (0.0)	1 (1.7)	0 (0.0)	[6]
	Unknown	1 (1.7)	4 (6.7)	1 (1.7)	[28, 30, 121, 122, 125, 128]
Algorithm for prediction of target	1-gram Term Frequency classifier	0 (0.0)	1 (2.4)	0 (0.0)	[44]
	Association rule mapping	0 (0.0)	1 (2.4)	0 (0.0)	[127]
	Chi-Square test	0 (0.0)	1 (2.4)	0 (0.0)	[126]
	Correlation analysis	0 (0.0)	2 (4.9)	0 (0.0)	[53, 119]
	Decision Tree	0 (0.0)	2 (4.9)	0 (0.0)	[45, 76]
	Fuzzy Algorithm for Extraction, Monitoring and Classification of infectious Diseases (FAEMC-ID)	0 (0.0)	1 (2.4)	0 (0.0)	[74]
	Hidden Markov Model	0 (0.0)	1 (2.4)	0 (0.0)	[123]
	*k*-Means clustering	1 (2.4)	1 (2.4)	0 (0.0)	[4, 120]
	*k*-Nearest Neighbors	1 (2.4)	0 (0.0)	0 (0.0)	[4]
	Latent Dirichlet allocation (LDA)	0 (0.0)	1 (2.4)	0 (0.0)	[44]
	Linear regression	0 (0.0)	5 (12.2)	0 (0.0)	[6, 28, 53, 125, 127]
	Maximum entropy	0 (0.0)	1 (2.4)	0 (0.0)	[125]
	Naïve Bayes	0 (0.0)	5 (12.2)	0 (0.0)	[24, 45, 76, 124, 125]
	Random Forest	0 (0.0)	1 (2.4)	0 (0.0)	[45]
	Recurrent neural networks with Long short-term memory (LSTM)	0 (0.0)	1 (2.4)	0 (0.0)	[53]
	ST-DBSCAN	0 (0.0)	1 (2.4)	0 (0.0)	[127]
	Support vector machines	1 (2.4)	8 (19.5)	1 (2.4)	[4, 24, 30, 44, 45, 49, 53, 58, 125, 128]
	Time series	0 (0.0)	1 (2.4)	0 (0.0)	[120]
	Unknown	1 (2.4)	2 (4.9)	0 (0.0)	[18, 121, 122]
Result	Negative	0 (0.0)	0 (0.0)	0 (0.0)	-
	Positive	2 (8.7)	20 (87.0)	1 (4.4)	[4, 6, 18, 24, 28, 30, 44, 45, 49, 53, 58, 74, 76, 119–128]
Reliability	Low	1 (4.4)	1 (4.4)	1 (4.4)	[18, 122, 128]
	Medium	1 (4.4)	15 (65.2)	0 (0.0)	[4, 24, 28, 30, 45, 49, 53, 58, 74, 76, 120, 121, 124–127]
	High	0 (0.0)	4 (17.4)	0 (0.0)	[6, 44, 119, 123]
Validity	Low	1 (4.4)	1 (4.4)	1 (4.4)	[18, 122, 128]
	Medium	1 (4.4)	15 (65.2)	0 (0.0)	[4, 24, 28, 30, 45, 49, 53, 58, 74, 76, 120, 121, 124–127]
	High	0 (0.0)	4 (17.4)	0 (0.0)	[6, 44, 119, 123]

Despite the overall comparability of the results, there are several notable and novel differences for the following themes: type of communicable disease and the quality of studies. The latter was operationalized as reliability and validity.

First, the studies that analyzed content from Twitter were most likely to investigate the communicable disease influenza (53.9%) [6, 18, 24, 28, 30, 44, 45, 49, 53, 58, 121, 123, 124, 126], followed by dengue (11.5%) [76, 125, 127] and measles (11.5%) [24, 74, 119]. Ebola [120], listeria [24], and tuberculosis [24] received far less attention (3.9% each), while HIV/AIDS was not at all investigated using content from Twitter.

Second, the quality of the included studies, which was measured as reliability and validity, overall was higher for publications that utilized content from Twitter. More specifically, half of the studies that used Sina Weibo [122] and all studies that utilized Yahoo! Knowledge [128] had low reliability and low validity, and relatively more studies that used Twitter were medium quality [24, 28, 30, 45, 49, 53, 58, 74, 76, 120, 121, 124–127]. In addition, all studies that were evaluated to be high quality also analyzed content from Twitter [6, 44, 119, 123].

## 5 Discussion

Overall, our results indicate that textual content from social media can be used reliably to monitor and surveille communicable diseases and to predict the trends of these diseases. This consistency of the evidence indicates that text mining of social media content may be a powerful and novel tool for public health authorities [28, 30]. This proactive and real-time tool addresses most of the limitations that are common among the traditional methods used for public health surveillance [1, 20, 21, 47, 50, 54]. In addition, this tool can be used for the remote sensing of user-generated experiences that were published to social media [19, 45, 57, 58]. This finding is consistent with the literature, which suggests that text mining of social media content has the potential to supplement the traditional methods for public health surveillance, such as the reporting of diagnosed cases by medical professionals [1, 45, 46].

Furthermore, Twitter was used most frequently as a source of user-generated health content. This finding is consistent with other studies that indicated that users publicly publish their own health-related information to Twitter, making Twitter a relevant social media platform [26, 27, 129, 130]. Some studies indicate, however, that Twitter may not be a reliable source for health-related content, and alternative sources should be identified that include content from this population [125].

Various techniques were used to process textual content. For example, sentiment analysis can be used to establish the subjectivity of content, such that news, which contains predominantly facts, can be distinguished from personal experiences that contain opinions. Because the included publications predominantly studied personal experiences and, therefore, excluded news, sentiment analysis or perhaps alternative strategies were used to classify this content.

Last, a discussion of our findings would not be complete without a reflection on Google Flu Trends. With the emergence of the internet, novel applications have been developed that collect and analyze data for the purpose of public health surveillance [19]. To address some of the challenges of the traditional methods for public health surveillance, the software company Google built Google Flu Trends, which utilizes influenza-related search queries and search patterns from its users to estimate regional seasonal influenza outbreaks [6, 131, 132]. The underlying presumption of using search queries to predict influenza is that people, when they experience changes in their health status, search the internet for symptoms, treatments, and other medical advice for self-diagnosis [50]. The influenza-related search queries may then be analyzed for early indications of a seasonal influenza outbreak [19]. Therefore, increases or decreases in these search patterns may indicate the outbreak or the end of the seasonal flu season, respectively [19]. This made Google Flu Trends a novel real-time and global tool for remote sensing [123]. To enable researchers and public health authorities to perform their own analyses, Google also publishes these historical datasets online [58].

Some studies reported that Google Flu Trends achieves a higher accuracy for the prediction of seasonal influenza outbreaks than traditional methods [15]. For example, these search queries were used to predict seasonal influenza rates two weeks in advance at a 90 percent accuracy [127]. Similarly, influenza-related hospital visits were also analyzed using Google Flu Trends [133].

However, many researchers have reported that Google Flu Trends still faces many drawbacks related to its accuracy [58, 124]. For example, Google Flu Trends was found to be inaccurate with respect to variations in seasonal influenza patterns that occur on an annual basis [134]. In addition, it did not predict the 2009 A(H1N1) pandemic and performed suboptimal in forecasting subsequent seasonal influenza seasons [134–138]. Predominantly, the reliability of Google Flu Trends has been seriously questioned since 2013, when it failed to predict the intensity of the seasonal influenza outbreak [139]. Others have also reported that that Google Flu Trends has suboptimal performance [140].

Although Google Flu Trends has remediated several limitations of traditional health surveillance methods, additional innovations that provide improvements are required to enable better public health surveillance [6]. Furthermore, due to the repeated failure to detect infectious disease outbreaks and the shortcomings described above, Google Flu Trends was discontinued in 2015 [54]. Therefore, there exists a need for alternative and more suitable surveillance methods [134, 140], which we aimed to address using the present systematic review.

### 5.1 Limitations

This systematic review had six noteworthy limitations.

First, study selection, information extraction, quality assessment of publications, and analysis were performed by one researcher (PP). This may have introduced bias. However, the procedures and results were discussed by all authors, and disagreements were resolved by consensus.

Second, in the included publications, there was an unequal distribution of the analyzed communicable diseases. For example, studies most often reported on the effectiveness of using social media to monitor and surveille influenza, while fewer studies analyzed the effectiveness in relation to dengue and measles. Likewise, Ebola, HIV/AIDS, listeria, and tuberculosis received the least attention. Therefore, most studies reported findings on the same diseases, but it remains unknown to what extent these positive findings also hold for infectious diseases that were studied least often.

Third, Twitter was investigated in a vast majority of studies. However, Sina Weibo and Yahoo! Knowledge received very little attention. Additionally, other social media platforms exist, such as Facebook, which were not investigated at all. Therefore, it remains unknown whether content from other source media platforms can also be used effectively for the public health surveillance of communicable diseases, especially because these platforms may be targeted to different populations and, thus, may enable the monitoring of specific subgroups in this population.

Fourth, it is common and unavoidable that user-generated content published to social media is inherently noisy and biased. Most users are unqualified to assess their medical symptoms and may exaggerate mild or unrelated symptoms. Users may also be malicious and intentionally publish fake content and seek to discredit competition. We suggest the consideration of these factors when evaluating the effectiveness of the data sources and proposed tools.

Fifth, a majority of the publications structurally failed to report important information. For example, many publications did not explicitly disclose the language and geographical origin of the included content, although this could sometimes be implicitly inferred. This is particularly relevant because a vast majority of studies used Twitter, which does record the geographical location of its users. Similarly, the software, as well as specific methods and techniques used for natural language processing, were often omitted. In addition to a lack of information about the implementation in the included studies, the authors often failed to reflect on their collaboration with the authorities, such as public health institutes. All studies investigated how text can be processed and understood, and the reporting of such crucial information is, therefore, essential for replicability.

Sixth, this qualitative systematic review followed the PRISMA guidelines. As discussed in the methodology section, due to the interdisciplinary nature of the reviewed studies and their limitations, we acknowledge that it was not possible to complete every item from the PRISMA checklist (see S2 Appendix). For the same reason, no PROSPERO registration was made.

### 5.2 Theoretical recommendations

In the following, four recommendations are suggested to researchers.

First, although a vast number of the researchers included in this study investigated influenza, which clearly makes influenza a popular disease on this topic, and to a lesser extent dengue and measles have also been studied, it is essential that other communicable diseases also receive more attention in the literature. Indeed, many infectious diseases exist that pose a threat to public health, and it remains unknown whether these diseases can be monitored and predicted effectively using textual content. Therefore, we recommend that other infectious diseases be studied more frequently to produce more evidence on this topic.

Second, similarly, Twitter is clearly a popular social media platform for text mining. Some of its popularity is also related to the public accessibility of its content. However, many other popular platforms exist that have received far less or even no attention in the literature. It is, therefore, recommended that future research also account for those platforms. This is particularly relevant because only then can it be established whether certain platforms are more useful than others to surveille and predict infectious diseases, or perhaps these platforms may yield contradicting findings.

Third, a majority of studies failed to report critical information, such as the language and geographical origin of their content and the software, methods, and techniques used for natural language processing. Including such information is essential to establish the reliability and validity of findings and because it enables other researchers to replicate the study. It is, therefore, recommended that researchers disclose such information. In addition, it is highly recommended that the software that was developed to collect and analyze the data in studies is well documented and published for reuse by the community and that authors thoroughly describe the application of their NLP analysis.

Fourth, the included journal articles were overall of higher quality than conference proceedings. This difference may be partly explained by the peer review involved, which may be more elaborate for journals than for conferences. However, another explanation is related to the limited amount of important information that was disclosed about the included data, methodologies, and analyses. Therefore, it is highly recommended that researchers provide more of the information needed to establish the reliability and validity of their studies and the reported findings.

## 6 Conclusion

Our findings in this work indicate that text mining of health-related content published to social media can serve as a novel and powerful tool for the automated, real-time, and remote monitoring of public health and for the surveillance and prediction of communicable diseases in particular.

According to our results, practitioners at public health authorities may benefit from utilizing natural language processing applied to social media data for the surveillance of communicable diseases as a supplement to their traditional methods. Natural language processing provides an automated, real-time tool to analyze user-generated content that includes contextual information to surveille and predict communicable diseases worldwide. This systematic review indicates that textual content from social media can be an important source of this knowledge. Another benefit of social media content is that it enables remote sensing via the internet by collecting public information. There is, however, no need to replace traditional methods, such as the collection of information about diagnosed cases from medical practitioners. Nevertheless, practitioners are highly recommended to include textual content from social media as a supplementary source for their data in their public health surveillance efforts to monitor and predict communicable diseases.

## Supporting information

S1 Appendix Search queries. (DOCX)

S2 Appendix PRISMA checklist. (DOCX)

S3 Appendix Characteristics of studies. (DOCX)
