# Supplementary material for: Surveillance of communicable diseases using social media: A systematic review
Source: PLoS One. 2023 Feb 24;18(2):e0282101. doi: 10.1371/journal.pone.0282101 (PMC9956027; doi:10.1371/journal.pone.0282101)
Supplement: S1 Appendix — (DOCX) [file pone.0282101.s001.docx]

Appendix 1

Table 4. Search query for ACM Digital Library

| Block 1: Natural Language Processing |
| --- |
| artificial intelligence OR machine learning OR text mining OR computational linguistics OR natural language processing OR nlp OR sentiment analysis OR word embedding* OR natural language toolkit OR nltk |
|  |
| Block 2: Public Health Monitoring |
| public health surveillance OR health surveillance OR public health monitoring OR health monitoring |
|  |
| Filters: |
| Searched within: Abstract, Author Keyword, Title  All publications: Proceedings, Research Article |

Table 5. Search query for IEEE Xplore

| Block 1: Natural Language Processing |
| --- |
| "All Metadata":artificial intelligence OR "All Metadata":machine learning OR "All Metadata":text mining OR "All Metadata":computational linguistics OR "All Metadata":natural language processing OR "All Metadata":nlp OR "All Metadata":sentiment analysis OR "All Metadata":word embedding* OR "All Metadata":natural language toolkit OR "All Metadata":nltk |
|  |
| Block 2: Public Health Monitoring |
| "All Metadata":public health surveillance OR "All Metadata":health surveillance OR "All Metadata":public health monitoring OR "All Metadata":health monitoring |
|  |
| Filters: |
| Document types: Conferences, Journals |

Table 6. Search query for PubMed

| Block 1: Natural Language Processing |
| --- |
| artificial intelligence[Title/Abstract] OR Artificial Intelligence[MeSH Terms] OR machine learning[Title/Abstract] OR Machine Learning[MeSH Terms] OR text mining[Title/Abstract] OR computational linguistics[Title/Abstract] OR natural language processing[Title/Abstract] OR Natural Language Processing[MeSH Terms] OR nlp[Title/Abstract] OR sentiment analysis[Title/Abstract] OR word embedding*[Title/Abstract] OR natural language toolkit[Title/Abstract] OR nltk[Title/Abstract] |
|  |
| Block 2: Public Health Monitoring |
| public health surveillance[Title/Abstract] OR Public Health Surveillance[MeSH Terms] OR health surveillance[Title/Abstract] OR public health monitoring[Title/Abstract] OR health monitoring[Title/Abstract] |
|  |
| Filters: |
| Article types: Journal Article  Languages: English |

Table 7. Search query for Web of Science

| Block 1: Natural Language Processing |
| --- |
| TS=(artificial intelligence) OR TS=(machine learning) OR TS=(text mining) OR TS=(computational linguistics) OR TS=(natural language processing) OR TS=(nlp) OR TS=(sentiment analysis) OR TS=(word embedding*) OR TS=(natural language toolkit) OR TS=(nltk) |
|  |
| Block 2: Public Health Monitoring |
| TS=(public health surveillance) OR TS=(health surveillance) OR TS=(public health monitoring) OR TS=(health monitoring) |
|  |
| Filters: |
| Document types: Article, Proceedings Paper  Languages: English |
