# Supplementary material for: Surveillance of communicable diseases using social media: A systematic review
Source: PLoS One. 2023 Feb 24;18(2):e0282101. doi: 10.1371/journal.pone.0282101 (PMC9956027; doi:10.1371/journal.pone.0282101)
Supplement: S3 Appendix — (DOCX) [file pone.0282101.s003.docx]

Appendix 3

Table 8. Characteristics of Studies Analyzed in Systematic Review (23 Studies Included)

| Authors | Publication type | Communicable disease | Social media platform | Sample size | Language of data | Period of data collection | Horizon of data collection | Country | Software for NLP | Processing for NLP | Target | Algorithm for prediction of target | Result | Description of result | Reliability | Validity |
| --- | --- | --- | --- | --- | --- | --- | --- | --- | --- | --- | --- | --- | --- | --- | --- | --- |
| [6] | Journal article | Influenza | Twitter | 1,089,684 tweets | - Arabic (518,558 tweets) - English (571,126 tweets) | - From November 1 to November 20, 2016 - From January 1 to January 30, 2017 | 2 months | United Arab Emirates | Natural Language Toolkit | - Language categorization - Stemming - Stop word removal - Tokenization - Tweet filtering | Number of seasonal influenza-related hospital visits | Linear regression | Positive | Future hospital visits related to seasonal influenza can be reliably predicted using tweets. | High | High |
| [58] | Journal article | Influenza | Twitter | Unknown | Unknown | From 2013 to 2014 | Unknown | United States | Unknown | Term Frequency - Inverse Document Frequency (TF-IDF) | Monitoring outbreaks of seasonal influenza | Support vector machines | Positive | Tweets have great potential to monitor outbreaks of seasonal influenza. | Medium | Medium |
| [49] | Journal article | Influenza | Twitter | 159,802 tweets | Unknown | From September 29, 2013 to March 1, 2014 | 6 months | United States | Unknown | Term Frequency - Inverse Document Frequency (TF-IDF) | Surveillance of seasonal influenza | Support vector machines | Positive | Tweets can be used to surveille seasonal influenza. | Medium | Medium |
| [124] | Conference proceeding | Influenza | Twitter | 4,696 tweets | English | From October 27 to November 30, 2015 | 1 month | Canada | - Apache Lucene’s PorterStemFilter - Apache Lucene’s StopFilter - OpenNLP - Stanford CoreNLP | - Detecting URLs - Homogenization - Sentiment analysis - Stemming - Stop word removal | Monitoring and prediction of the spread of seasonal influenza | Naïve Bayes | Positive | Tweets can be used to monitor and predict the spread of seasonal influenza with high accuracy. | Medium | Medium |
| [123] | Journal article | Influenza | Twitter | ± 6.1 million tweets | Unknown | From December 2012 to August 2014 | 21 months | Unknown | Datasift service | - Lemmatization - Stemming | Prediction of seasonal influenza trends | Hidden Markov Model | Positive | Tweets can be used to accurately predict seasonal influenza trends. | High | High |
| [126] | Journal article | Influenza | Twitter | More than 2 million tweets | English | From May 1 to December 31, 2009 | 8 months | Unknown | Unknown | Sentiment analysis | Trends in increase of H1N1-related tweets | Chi-Square test | Positive | Tweets can be used for real-time monitoring of H1N1. | Medium | Medium |
| [127] | Conference proceeding | Dengue | Twitter | 493,102 tweets (first dataset: 27,658; second dataset: 465,444) | Unknown | - From January to July 2009 - From December 2010 to April 2011 | - 7 months - 5 months | Brazil | Unknown | Sentiment analysis | Prediction of dengue incidence for each city | - Association rule mapping - Linear regression - ST-DBSCAN | Positive | Tweets can be used to predict denge epidemics both spacially and temporally. | Medium | Medium |
| [74] | Journal article | Measles | Twitter | 2,870 tweets | Unknown | From March 1 to March 8, 2019 | 1 week | Unknown | Unknown | - Homogenization - *n*-gram generation - Stemming - Stop word removal - Term filtering - Term Frequency - Inverse Document Frequency (TF-IDF) - Tokenization | Monitoring of measles | Fuzzy Algorithm for Extraction, Monitoring and Classification of infectious Diseases (FAEMC-ID) | Positive | Tweets and news on social media can be used to monitor measles. | Medium | Medium |
| [45] | Journal article | Influenza | Twitter | 730 tweets | Unknown | Unknown | Unknown | India | Unknown | - Dimensionality reduction - Feature weighting - Lemmatization - Normalization using frequency-based methods - Stemming - Stop word removal - Tokenization | Identification of suspected cases of H1N1 | - Decision Tree - Naïve Bayes - Random Forest - Support vector machines | Positive | Tweets can be used effectively to identify suspected cases of H1N1. | Medium | Medium |
| [24] | Conference proceeding | - Influenza  - Listeria - Measles - Tuberculosis | Twitter | Unknown | Unknown | From October 18, 2011 to April 6, 2012 | 6 months | United States | Unknown | - Sentiment analysis - Stemming - Term Frequency - Inverse Document Frequency (TF-IDF) - Unigram generation | Identification and monitoring of negative about infectious diseases | - Naïve Bayes - Support vector machines | Positive | Tweets can be used to monitor sentiment about infectious diseases. | Medium | Medium |
| [44] | Journal article | Influenza | Twitter | 40,428 tweets | English | From August 31, 2012 to March 4, 2013 | 6 months | United States | - Stanford CoreNLP - The Stanford parser | - Lemmatization - Term Frequency - Inverse Document Frequency (TF-IDF) - Unigram generation | Seasonal influenza surveillance using consumed drugs | - 1-gram Term Frequency classifier - Latent Dirichlet allocation (LDA) - Support vector machines | Positive | Tweets can be used to extract drugs consumption to enhance seasonal influenza surveillance. | High | High |
| [128] | Conference proceeding | HIV/AIDS | Yahoo! Knowledge | 670 forum messages | Unknown | From January 1, 2007 to September 20, 2009 | 33 months | Taiwan | Unknown | Unknown | Surveillance of HIV/AIDS | Support vector machines | Positive | Content on health-focused web forums can be used to surveille HIV/AIDS. | Low | Low |
| [18] | Conference proceeding | Influenza | Twitter | 6,097,406 tweets | Unknown | Unknown | 6 months | Unknown | Unknown | - Stop word removal - Tokenization | Real-time surveillance of seasonal influenza outbreaks | Unknown | Positive | Tweets can be used for real-time surveillance of seasonal influenza outbreaks. | Low | Low |
| [119] | Journal article | Measles | Twitter | 20,201 tweets | Unknown | From April 15 to November 11, 2013 | 7 months | The Netherlands | Unknown | - Sentiment analysis - Thematic analysis | Detection of disease patterns of measles | Correlation analysis | Positive | Tweets can be monitored for public opinion patterns to detect disease patterns of measles. | High | High |
| [28] | Journal article | Influenza | Twitter | 2,972 tweets | Unknown | From October 15, 2012 to May 10, 2013 | 6 months | United States | Unknown | Unknown | Prediction of daily emergency department visits for influenza-related illnesses | Linear regression | Positive | Tweets can be used to predict daily emergency department visits for influenza-related illnesses. | Medium | Medium |
| [125] | Journal article | Dengue | Twitter | 16,818 tweets | Unknown | From October 2012 to December 2014 | 27 months | Brazil | Unknown | Unknown | Spatial and temporal reporting trends for dengue | - Linear regression - Maximum entropy - Naïve Bayes - Support vector machines | Positive | Tweets can be used to identify cases of dengue. | Medium | Medium |
| [120] | Journal article | Ebola | Twitter | 42,236 tweets | English | From July 24 to August 1, 2014 | 1 week | Unknown | Unknown | - Content analysis - *n*-gram generation - Remove symbols and URLs - Topic detection | Real-time detection of Ebola outbreak and monitoring its spread | - *k*-Means clustering - Time series | Positive | Tweets can be used for real-time detection of Ebola outbreak and to monitor its spread. | Medium | Medium |
| [121] | Conference proceeding | Influenza | Twitter | 81,236 tweets | Unknown | From July 1, 2013 to September 30, 2014 | 15 months | - Australia - New Zealand | Unknown | Unknown | Detection of seasonal influenza outbreak | Unknown | Positive | Tweets can be used to detect seasonal influenza outbreaks. | Medium | Medium |
| [76] | Conference proceeding | Dengue | Twitter | 667 tweets | Unknown | From January 2009 to October 2017 | 106 months | Brazil | Unknown | - *n*-gram generation - Stemming - Stop words removal - Term Frequency - Inverse Document Frequency (TF-IDF) | Detection of dengue epidemics | - Decision Tree - Naïve Bayes | Positive | Tweets can be used to identify dengue epidemics. | Medium | Medium |
| [53] | Journal article | Influenza | Twitter | 171,027,275 tweets | Unknown | From January 2011 to December 2014 | 48 months | United States | Unknown | - LDA topics - *n*-gram generation - Term Frequency - Inverse Document Frequency (TF-IDF) - Text embeddings | Geographical prediction of seasonal influenza cases | - Correlation analysis - Linear regression - Recurrent neural networks with Long short-term memory (LSTM) - Support vector machines | Positive | Tweets can be used to predict seasonal influenza in various geographical locations. | Medium | Medium |
| [30] | Journal article | Influenza | Twitter | 7,666,201 tweets | Japanese | From August 2, 2012 to March 1, 2016 | 43 months | Japan | Unknown | Unknown | Detection of seasonal influenza | Support vector machines | Positive | Tweets can be used to predict seasonal influenza. | Medium | Medium |
| [4] | Conference proceeding | Influenza | Sina Weibo | 3,505,110 posts | Mandarin | From September to December 2013 | 4 months | China | Unknown | Term Frequency - Inverse Document Frequency (TF-IDF) | Prediction and detection of seasonal influenza outbreak | - *k*-Means clustering - *k*-Nearest Neighbors - Support vector machines | Positive | Content from Sina Weibo can be used to predict and detect the outbreak of seasonal influenza. | Medium | Medium |
| [122] | Journal article | Influenza | Sina Weibo | 718,419 posts | Mandarin | From March 31 to April 30, 2013 | 1 month | China | Unknown | Unknown | Monitoring of Avian influenza A (H7N9) outbreak | Unknown | Positive | Content from Sina Weibo can be used to surveille the Avian influenza A (H7N9) outbreak | Low | Low |
